# Enhancing bladder cancer diagnosis through transitional cell carcinoma polyp detection and segmentation: an artificial intelligence powered deep learning solution

**DOI:** 10.3389/frai.2024.1406806

**Published:** 2024-05-30

**Authors:** Mahdi-Reza Borna, Mohammad Mehdi Sepehri, Pejman Shadpour, Farhood Khaleghi Mehr

**Affiliations:** ^1^Department of IT Engineering, Faculty of Industrial and Systems Engineering, Tarbiat Modares University, Tehran, Iran; ^2^Hasheminejad Kidney Center (HKC), Iran University of Medical Sciences, Tehran, Iran

**Keywords:** image-guided surgery, segmentation, modelling, computer-aided diagnosis, TCC tumors, cystoscopy

## Abstract

**Background:**

Bladder cancer, specifically transitional cell carcinoma (TCC) polyps, presents a significant healthcare challenge worldwide. Accurate segmentation of TCC polyps in cystoscopy images is crucial for early diagnosis and urgent treatment. Deep learning models have shown promise in addressing this challenge.

**Methods:**

We evaluated deep learning architectures, including Unetplusplus_vgg19, Unet_vgg11, and FPN_resnet34, trained on a dataset of annotated cystoscopy images of low quality.

**Results:**

The models showed promise, with Unetplusplus_vgg19 and FPN_resnet34 exhibiting precision of 55.40 and 57.41%, respectively, suitable for clinical application without modifying existing treatment workflows.

**Conclusion:**

Deep learning models demonstrate potential in TCC polyp segmentation, even when trained on lower-quality images, suggesting their viability in improving timely bladder cancer diagnosis without impacting the current clinical processes.

## 1 Introduction

Bladder cancer is the the10th most common form of cancer worldwide (Wu et al., 2022). More than 80,000 new cases of bladder cancer and 17,000 deaths occurred in the United States in 2018 (Shkolyar et al., 2019). In addition, the data in China indicate that bladder cancer ranked sixth in male cancer incidence in 2015 (Pang et al., 2016). Five thousand eight hundred seventeen, five thousand six hundred sixty-two, and six thousand six hundred thirty new bladder cancer cases in 2014, 2015 and 2016, respectively were registered in Iran (Partovipour et al., 2022). Transitional cell carcinoma (TCC) is the most common type of bladder cancer by far. Approximately 90 percent of bladder cancers are TCC (Willis and Kamat, 2015).

Diagnostic and therapeutic cystoscopy is crucial for bladder cancer diagnosis and treatment. There are a lot of studies that address treatment and diagnosis of bladder cancer (Doluoglu et al., 2022; Larizadeh et al., 2023; Pazır et al., 2023). Transurethral Resection of Bladder Tumors (TURBT) is performed for the pathological diagnosis and staging of patients with suspicious bladder lesions detected during cystoscopy. Almost all papillary and sessile bladder tumors can be detected with cystoscopy, which is the gold standard of bladder tumor detection (van der Aa et al., 2010). Detecting a bladder tumor is crucial for reducing the likelihood of cancer recurrence following complete transurethral resection. As a result, accurate identification of bladder tumors under cystoscopy is of major clinical significance. Urologists can miss bladder tumors, resulting in misdiagnosis and incomplete TURBT (Brausi et al., 2002). Also the surgeon’s visual perception of high grade TCC is the basis for beginning costly intraoperative chemotherapeutic instillation. Therefore, using computer-aided tools to detect tumors during cystoscopy may be helpful (Ferro et al., 2023).

The rapid expansion of artificial intelligence and machine learning methods across various medical domains, particularly in emergency medicine, is evident (Shafaf and Malek, 2019) and there exist some studies in the field of bladder cancer, computer-aided detection of TCC tumors in cystoscopy images is challenging, and previous research has been quite limited. Wu et al. (2022) report a method called Cystoscopy Artificial Intelligence Diagnostic System (CAIDS) diagnosis for bladder cancer. Their study collected 69,204 images of 10,729 consecutive patients from 6 hospitals and separated them into 69,204 training, internal, and external validation sets. The proposed method has been able to achieve a 95% sensitivity. Accordingly, Shkolyar et al. (2019) utilize a deep learning algorithm for importing cystoscopic detection of bladder cancer. In order to train and test an algorithm for automated bladder tumor detection. To this end a dataset of 95 patients for development was used. Additionally, 54 patients were prospectively evaluated for the proposed method called CystoNet’s diagnostic performance. The results show that the proposed method’s sensitivity increases by up to 90%. Ali et al. (2021) deployed blue light (BL) image-based artificial intelligence (AI) diagnostic platform using 216 BL images for the classification of cystoscopy images. By deploying a convolutional neural network (CNN) for image classification, the sensitivity and specificity of classification for malignant lesions were 95.77 and 87.84%, respectively. Ikeda et al. (2021), deployed a method based on transfer learning (TL) which trained a convolutional neural network with 1.2 million general images. To customize the TL for cystoscopy images, the TL-based model was trained with 2,102 cystoscopic images. The developed TL-based model had 95.4% sensitivity and 97.6% specificity. The performance of this model was better than that of the other models and comparable to the performance of expert urologists. Furthermore, it showed superior diagnostic accuracy when tumors occupied more than 10% of the image. By adopting the U-Net method, Yeung et al. (2021) study tubal diagnosis in colonoscopy methods. Obtained results show a 15*%* improvement over the previous works on the used data set.

Among all computer-aided methods, deep neural network (DNN), which exhibits brain-inspired characteristics, revolutionized artificial intelligence and has also shown great potential in computer-aided diagnostics in various fields such as radiology, histopathology, and computational neuroscience (Gao and Mosalam, 2018). Inspired by related work, we have compared U-Net-based methods that have achieved higher accuracy.

The rest of the paper presents the following sections: models for semantic segmentation are presented in section 2. In section 3, we describe how synthetic blastocyst images are generated and provide experimental results. The final section of this article concludes this research and suggests some directions for future research.

## 2 Proposed method

Knowledge of the global context is beneficial in semantic segmentation, if not essential. Adding more layers of convolutions and downsampling are two of the most common methods of increasing the receptive field of a convolutional network (Yamashita et al., 2018). A linear receptive field expansion is achieved with the first strategy, while a multiplicative expansion is achieved with the second. The U-Net is a modern architecture that combines both.

### 2.1 U-Net

In this study, the U-Net architecture, initially introduced by Ronneberger et al. (2015), is employed. Primarily designed for biomedical image segmentation, U-Net comprises two pathways known as the encoder and decoder. The encoder, also referred to as the contraction path, focuses on extracting feature maps and capturing image information using a conventional CNN architecture with convolution and max pooling layers. On the other hand, the decoder path, which is the distinctive feature of U-Net, merges feature and spatial information for localization (Sultana et al., 2020). This symmetric expanding path, also called the decoder, is designed to accommodate images of any size and thus excludes dense layers, containing only convolutional layers (Ambesange et al., 2023).

### 2.2 Other architectures

#### 2.2.1 LinkNet

Many segmentation architectures employ multiple downsampling operations, potentially leading to the loss of spatial information. Addressing this challenge, the LinkNet architecture, proposed by Chaurasia and Culurciello (2017), offers a solution by establishing connections between each encoder and its corresponding decoder. This approach facilitates the recovery of spatial information through upsampling operations, enhancing efficiency by reducing the parameters in the decoder (Sulaiman et al., 2024).

#### 2.2.2 MANet

Proposed by Xu et al. (2021) for classifying COVID-19 positive cases from chest X-ray images, the MANet architecture incorporates attention mechanisms to enhance network capabilities and performance. The attention block directs the model’s focus towards critical areas of the input image, ensuring that irrelevant features do not interfere with the training process—an essential capability for medical image analysis (Al Qurri and Almekkawy, 2023).

#### 2.2.3 U-Net++

U-Net++ presents an encoder-decoder architecture with several advantages over the traditional U-Net (Zhou et al., 2018). Recognizing that standard U-Net construction may result in detail loss, U-Net++ includes skip pathways between the encoder and decoder to mitigate this issue. Additionally, it integrates dense convolution blocks to bridge the gap between encoder and decoder feature maps. Another noteworthy feature is deep supervision, allowing the model to adapt its performance based on the context, balancing between accuracy and speed as required.

#### 2.2.4 PSPNet

**Figure 1:**
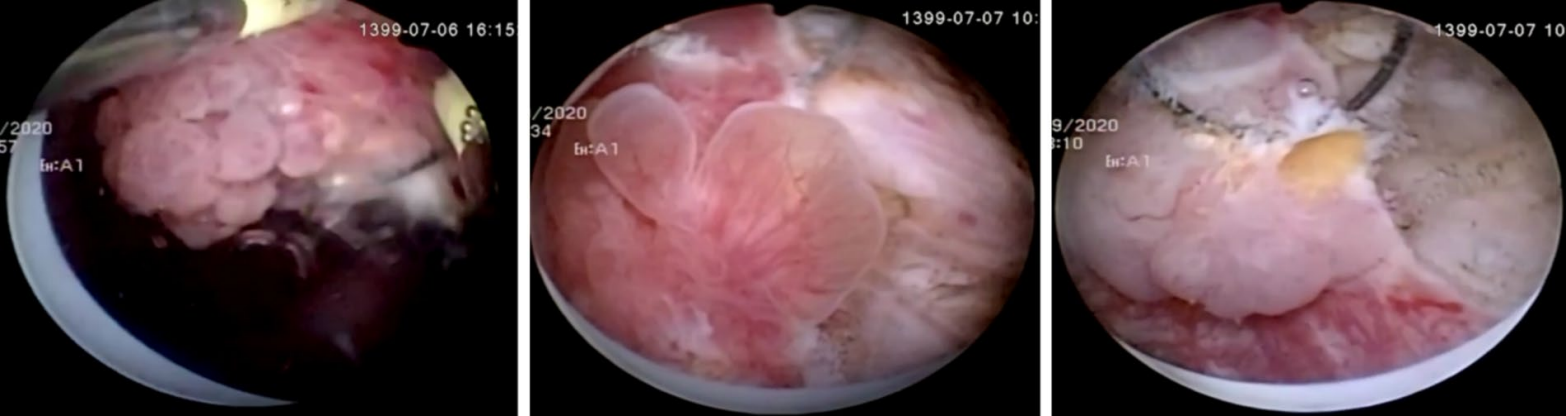
Samples of Transitional Cell Carcinoma high and low grade polypoid structures on cystoscopic images.

**Figure 2 fig2:**
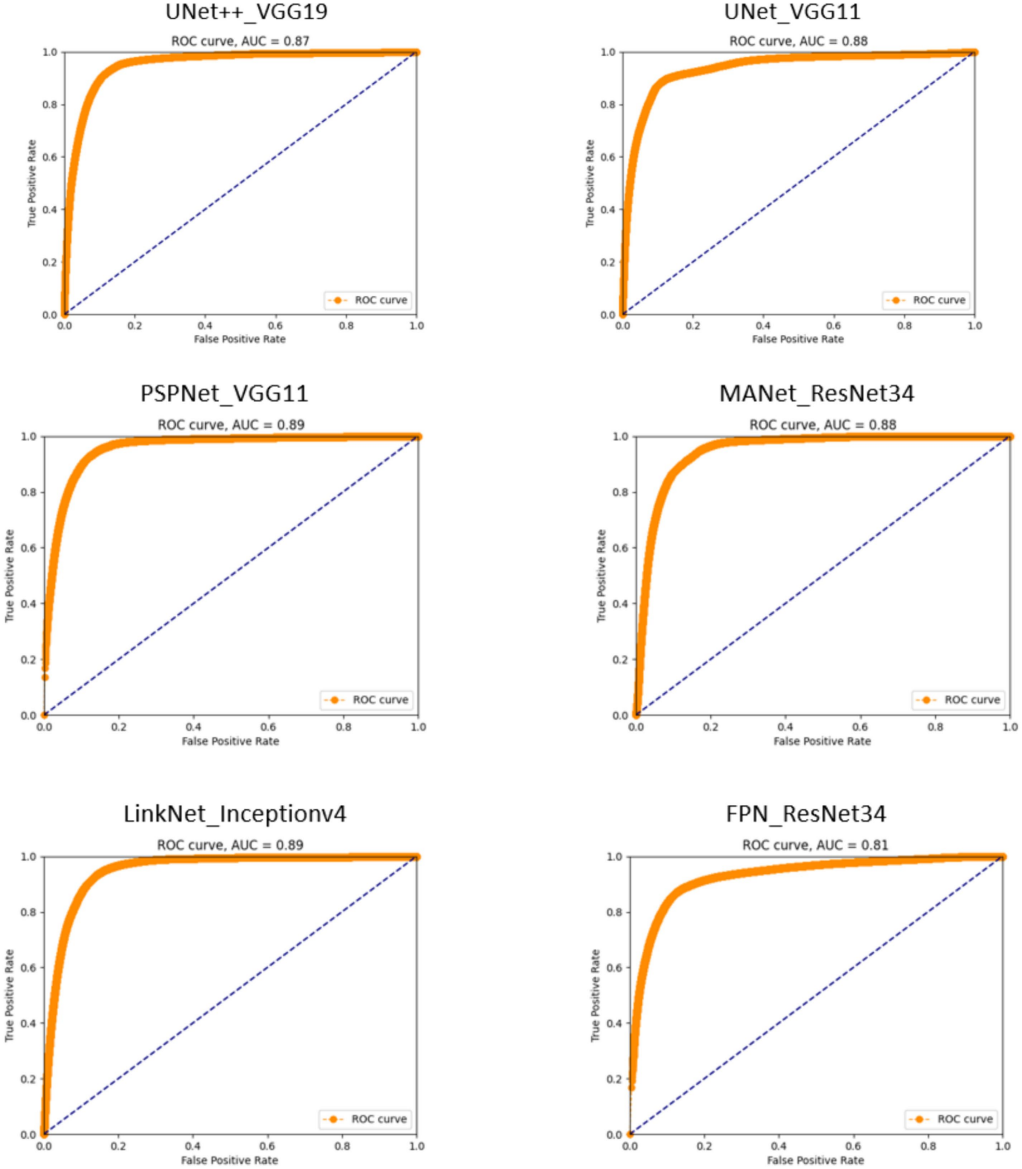
Receiver operating characteristic (ROC) curves for segmentation models. The proximity of each curve to the upper-left corner of the plot reflects the model’s effectiveness, with curves closer to this corner indicating superior performance.

**Figure 3:**
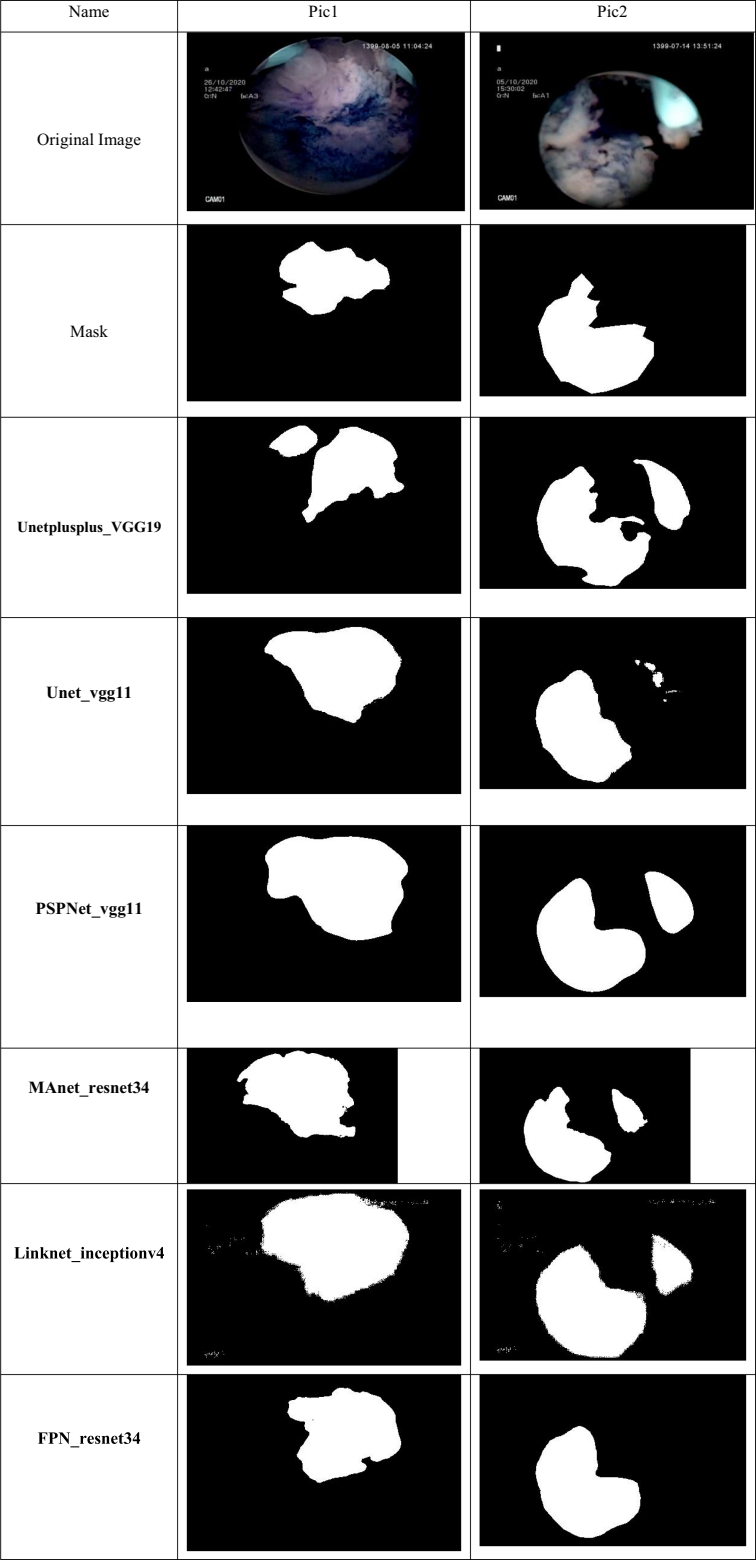
Samples of ground truth and predicted mask plus original images of twisted polyp images.

Initially introduced in the ImageNet scene parsing challenge 2016 by Zhao and Sun (2017), the Pyramid Scene Parsing Network (PSPNet) enhances global context comprehension in images. By replacing common convolutional layers with dilated convolutional layers, PSPNet effectively enlarges the respective field. The pyramid pooling module enables the model to pool feature maps across multiple scales, facilitating feature capture at various resolutions (see Figures 1 [Fig fig2]–3).

#### 2.2.5 FPN

Feature pyramids are adept at discerning objects at different scales, but their computational cost can be prohibitive. The Feature Pyramid Network (FPN), introduced by Chaurasia and Culurciello (2017), mitigates this issue by offering advantages over traditional pyramidal feature hierarchies without significantly increasing computational load. Comprising a bottom-up pathway for feedforward computation and a top-down pathway for up sampling higher resolution features, FPN connects these pathways via lateral connections to effectively integrate multi-scale information. The comparison of these models is presented in Table 1.

**Table 1:** Comparison of the architectures which are used in the segmentation task.

Feature/model	UNet	UNet++	FPN	LinkNet	PSPNet	MANet
Skip connections	Yes	Nested	Yes	Yes	No	No
Nested architecture	No	Yes	No	No	No	No
Feature pyramid	No	No	Yes	Yes	No	No
Multi-scale features	No	Yes	No	Yes	Yes	No
Attention mechanisms	No	No	No	No	No	Yes

## 3 Experimental results

### 3.1 Dataset

Data were retrospectively collected from 112 patients who had been previously diagnosed with TCC bladder cancer by TURBT at the Hasheminejad Hospital, Iran University of Medical Sciences, Tehran, Iran, from September 2020 to September 2021. Videos of their surgery were recorded and total of 1,058 cystoscopic images were collected from patients who were diagnosed with bladder cancer. Two hundred images were chosen and finally, each image has been annotated to distinguish tumor parts from the background. The study was approved by Research Ethics Committees of Tarbiat Modares University (IR.MODARES.REC.1401.107). The study followed the guidelines and protocols described in the routine practice of the IVF unit. No further interventions were used during treatment. During the data analysis, none of the authors had access to the patients’ information (see Table 2).

**Table 2:** Summary of data used in this study.

Section	Number of samples
Train	160
Augmented train	960
Test	40
Total	200

#### 3.1.1 Augmentation

Increasing the size of annotated datasets significantly impacts the performance of deep learning models, as these networks tend to excel when trained on extensive data. However, assembling such datasets poses challenges, given the costly and labor-intensive nature of image annotation (Chaitanya et al., 2021). To mitigate this issue, data augmentation emerges as a preferred strategy. This technique involves generating diverse versions of existing images within the dataset to expand the training set. We implemented various static augmentation methods on our dataset, employing the following techniques and their corresponding parameters:

##### 3.1.1.1 Rotation

– Parameter: rotation angle range (−15 to +15 degrees).
– Description: random rotations within a specified angle range (−15 to +15 degrees) were applied to each image, introducing rotational invariance and generating a diverse set of rotated versions.

##### 3.1.1.2 Flip (horizontal and vertical)

– Parameter: random horizontal and vertical flip.
– Description: images were randomly flipped both horizontally and vertically, each with a 50% probability. This augmentation increased dataset variability and facilitated object recognition regardless of orientation.

##### 3.1.1.3 Brightness and contrast adjustment

– Parameter: random brightness shift (−20 to +20), random contrast scaling (0.8 to 1.2).
– Description: random adjustments were made to brightness (with shifts ranging from −20 to +20) and contrast (scaling factors varied between 0.8 and 1.2) to simulate different lighting conditions.

##### 3.1.1.4 Gaussian noise

– Parameter: noise intensity (e.g., Mean = 0, Standard Deviation = 10).
– Description: Gaussian noise was introduced to a subset of images, with parameters such as a mean of 0 and a standard deviation of 10 controlling the intensity of noise.

##### 3.1.1.5 Color augmentation

– Parameters: random saturation (0.8 to 1.2) and Hue shift (−30 to +30 degrees).
– Description: random color transformations included saturation adjustments within the range of 0.8 to 1.2 and hue shifts ranging from −30 to +30 degrees, enabling handling of different color conditions effectively.

#### 3.1.2 Ground truth

The efficacy of the proposed methodology is appraised using ground truth data, meticulously delineated by expert urologists specializing in bladder cancer. The tumor boundaries are precisely demarcated with a one-pixel-wide contour, allowing for an accurate assessment of the model’s segmentation performance. Here are elaborations on TCC annotation process:

Number of raters: the annotations were conducted by three experienced urologists to ensure a robust and medically informed delineation of TCC polyps.

Mitigation of inter-rater variability: we employed several measures to ensure consistency across annotations:

- Initial training session to standardize the understanding of TCC characteristics.
- Regular consensus meetings to discuss and resolve any discrepancies in annotations.
- Statistical analysis of inter-rater reliability, achieving a Cohen’s kappa coefficient of 0.85, indicating substantial agreement.

### 3.2 Evaluation metrics

Precision, Recall, Accuracy, and Dice coefficient are the four measures we use to measure DeepCysto’s quality. Precision shows how precise the model is in the detection of positive samples (Equation 1).


(1) $$\mathrm{Precision}=\frac{TP}{TP+FP}$$

Recall indicates how many of the positive instances are identified by the algorithm (Equation 2).


(2) $$\mathrm{Recall}=\frac{TP}{TP+FN}$$

The Accuracy of the model refers to how well it performs across all classes of data (Equation 3).


(3) $$\mathrm{Accuracy}=\frac{TP+TN}{TP+TN+FP+FN}$$

In the context of this study, the Dice coefficient, also known as the Dice Similarity Coefficient (DSC), is employed as a critical metric to evaluate the efficacy of deep learning models in the segmentation of transitional cell carcinoma (TCC) polyps from cystoscopy images. This metric quantitatively assesses the similarity between the predicted segmentation masks generated by the models and the ground truth masks annotated by medical experts (Zou et al., 2004). By calculating the overlap twice, the area of intersection divided by the sum of the areas of both the predicted and true masks, the Dice coefficient offers a value between 0 and 1, where 1 signifies a perfect match and 0 denotes no overlap at all. This measure is particularly important in medical image analysis, as it directly relates to the models’ ability to accurately delineate areas of interest, crucial for the early diagnosis and treatment planning of bladder cancer.

In all the above equations, the TP indicates positive instances correctly identified as positive, and TN measures the number of negative instances correctly identified as negative. FP is the number of negative instances incorrectly identified as positive, and FN signifies the number of positive instances that are falsely missed.

### 3.3 Implementation details

The suggested deep learning architectures are deployed using an NVIDIA GeForce GTX 1070 featuring 8-gigabyte memory and 32-gigabyte RAM. The essential software packages for these models include Python 3.5 and Pytorch 1.2.0. The network takes inputs and produces outputs in the form of images with dimensions of 256 × 256 pixels. A soft variant of the Jaccard index, ensuring differentiability, serves as the loss function to minimize the disparity between the ground truth and the network’s predictions. Training involved 8 mini-batches, each comprising 25 samples, utilizing the Adam optimizer with an initial learning rate set to 0.0001.

### 3.4 Quantitative evaluation

#### 3.4.1 Models performance comparison

Table 3 provides a detailed comparison between the architecture of our proposed model and those of other deep learning models considered in this paper. In our comparative study of various U-Net-based models for the detection of TCC polyps in cystoscopy images, Unetplusplus_vgg19 showcased a promising precision of 55.40% and an impressive recall of 79.97%, achieving an accuracy of 92.32%. The model’s Dice coefficient of 80.57% indicates a reliable segmentation capability, although there is a noticeable shift from the traditional U-Net models previously reported higher precision.

**Table 3:** Results of different segmentation methods on cystoscopy images.

Method	Precision	Recall	Accuracy	Dice coefficient
Unetplusplus_vgg19	0.554032	0.799702	0.923155	0.805672
Unet_vgg11	0.496587	0.840718	0.907901	0.785945
PSPNet_vgg11	0.489003	0.878789	0.905354	0.787064
MAnet_resnet34	0.486019	0.849069	0.904505	0.781805
Linknet_inceptionv4	0.443667	0.891832	0.888333	0.763927
FPN_resnet34	0.574105	0.678503	0.924901	0.790132

The Unet_vgg11 and PSPNet_vgg11 models exhibited precision rates of 49.66 and 48.90%, respectively, with recall rates exceeding 84%, and accuracies just above 90%. These figures suggest that while these models are generally effective in polyp detection, there may be a greater inclusion of false positives within their segmentations.

The MAnet_resnet34 and Linknet_inceptionv4 models leaned towards higher recall rates of 84.91 and 89.18% but with lower precision, indicating a propensity to correctly identify polyps but also to misclassify healthy tissue as pathological. This is evidenced by the respective Dice coefficients of 78.18 and 76.39%.

Interestingly, the FPN_resnet34 model achieved the highest precision among the new set of models at 57.41%, yet its recall was the lowest at 67.85%. This suggests a conservative approach to polyp identification, prioritizing certainty over coverage, as reflected in its Dice coefficient of 79.01%.

These models’ performance illustrates the complex trade-offs between precision, recall, and accuracy in medical image segmentation. While no model uniformly outperformed the others across all metrics, each offers distinct strengths that could be advantageous depending on the specific clinical requirements. The FPN_resnet34’s precision, for example, might be preferred in scenarios where false positives carry a higher risk, while the Linknet_inceptionv4’s recall might be more desirable when it is crucial to detect as many cases as possible.

These findings highlight the potential of deep learning in medical diagnostics, where the choice of architecture plays a crucial role. The results also emphasize the need for a nuanced approach to model selection, tailored to the unique demands of medical image analysis, to ensure that the deployment of these technologies in clinical settings is both effective and reliable.

#### 3.4.2 Analyzing time and size of models

Table 4 presents a detailed comparison of the models used in our study, focusing on their computational aspects. Model size, measured in the number of parameters, and average segmentation time, measured in milliseconds, are critical metrics for assessing the feasibility of these models in clinical settings, particularly when real-time image processing is required.

**Table 4:** Comparison of model size and average segmentation time on a batch of test images in milliseconds.

Method	Model size	Average segmentation time (ms)
Unetplusplus_vgg19	44,655,889	87.05
Unet_vgg11	18,252,881	51.85
PSPNet_vgg11	10,012,289	37.28
MAnet_resnet34	31,777,361	65.15
Linknet_inceptionv4	46,158,561	91.78
FPN_resnet34	23,149,121	59.33

The model size is an indicator of the complexity and computational resource requirements of each model, while the average segmentation time provides insight into the model’s performance speed in processing individual images. These metrics are vital for understanding both the potential computational burden in clinical implementations and the practicality of using these models in scenarios where quick decision-making is crucial, such as during live cystoscopic examinations. The comparative data enable clinicians and researchers to make informed decisions regarding the selection of an appropriate model based on their specific operational and resource environments.

#### 3.4.3 ROC curves

The ROC curves delineate the distinct capabilities of each model, creating a visual narrative that aligns with the quantified metrics of precision, recall, and accuracy. The Unetplusplus_vgg19 model with an AUC of 0.87 and the Unet_vgg11 model with an AUC of 0.88 suggest that both models are competent in classifying TCC polyps, although there is an indication that they could be further refined to enhance their specificity, as inferred from their precision rates of 55.40 and 49.66%, respectively.

The PSPNet_vgg11 model’s ROC curve, which achieves an AUC of 0.89, reflects its strong performance in maintaining a commendable true positive rate, despite a precision rate that suggests potential for improvement in differentiating between polyp and non-polyp regions. This is indicative of a model that prioritizes sensitivity over precision.

The MANet_resnet34 model displays a similar trend with an AUC of 0.88, which, in concert with its precision rate of 48.60%, signals a solid ability in identifying polyps but also points towards a propensity to include false positives within its predictions.

Conversely, the Linknet_inceptionv4 model, while achieving a high recall rate, exhibits a lower precision of 44.37%, as reflected in its AUC of 0.89. This underscores a model that is adept at capturing most polyps at the expense of increased false positives.

The FPN_resnet34 model, although recording the lowest AUC at 0.81, indicates a nuanced performance. It achieves the highest precision rate among the models at 57.41%, suggesting that when it identifies polyps, it does so with a high degree of confidence, but may miss some true positives, a characteristic that could be favorable in certain clinical scenarios where false positives are a significant concern.

Overall, these ROC curves and their corresponding AUC values illustrate a spectrum of performance across the models, with none showing absolute dominance in all metrics. The results emphasize the importance of selecting a model that aligns with the specific needs of the clinical application, considering the balance between sensitivity and specificity. While the AUC values have varied, the potential of these models in detecting TCC polyps within cystoscopy images is evident, indicating their viable application in medical diagnostics with appropriate consideration for the inherent trade-offs each model presents.

#### 3.4.4 Sample segmentations of models

The performance of the various deep learning models applied to cystoscopy images for polyp detection is visually represented in the comparative analysis of predicted masks against the true mask. The true mask serves as the ground truth, and the proximity of a model’s predicted mask to this true mask is indicative of its accuracy and precision.

The Unet_vgg11 model shows a reasonable approximation to the true mask, albeit with some regions of over-segmentation, particularly noticeable in the second sample. This is consistent with the model’s higher recall rate, indicating a tendency to capture most of the polyps at the expense of precision.

PSPNet_VGG11’s predicted masks suggest a similar trend of over-segmentation, with the second sample showing significant deviation from the true mask. This observation aligns with the model’s high recall but lower precision, which may lead to more false positives in clinical settings.

MANet_ResNet34 exhibits a more conservative approach in the first sample, closely mirroring the true mask. However, in the second sample, the model demonstrates under-segmentation, suggesting a potential limitation in capturing the full extent of the polyps, as reflected by its balance between precision and recall.

Linknet_inceptionv4’s predicted masks diverge considerably from the true mask in both samples, with notable under-segmentation in the first sample and over-segmentation in the second. The high recall rate and lower precision of this model may account for these discrepancies, indicating a model less adept at precise boundary delineation.

FPN_ResNet34 presents a predicted mask that closely aligns with the true mask in the first sample, indicating strong performance. In contrast, the second sample shows under-segmentation, suggesting that while the model has high precision, it may struggle to detect all relevant polyps, which could lead to false negatives.

Unetplusplus_VGG19, while not shown in the provided analysis image, would be expected to display a predicted mask with a balance of precision and recall. Given its overall performance metrics, we anticipate a more consistent alignment with the true mask than some other models.

The analysis of these predicted masks emphasizes the necessity of a tailored approach when selecting models for clinical use. Models with higher precision may be preferable in scenarios where false positives present significant risks, while those with higher recall may be better suited for cases where the detection of every possible polyp is critical. The trade-offs highlighted by the mask predictions underscore the importance of considering both the quantitative metrics and the visual assessment of model performance in medical imaging tasks.

#### 3.4.5 Performance analysis under variable brightness and contrast

In clinical practice, cystoscopy images can exhibit significant variability in brightness and contrast due to differences in equipment settings, lighting conditions, and patient-specific factors. To ensure the robustness of our models under such varying conditions, we conducted a series of tests to evaluate the performance of our best-performing model, FPN_resnet34, under altered brightness and contrast levels.

The brightness of the images was adjusted by ±20% and the contrast by ±15% using linear scaling. These adjustments mimic common variations encountered in clinical settings. We then measured the segmentation performance of the FPN_resnet34 model on these modified images, focusing on precision, recall, and Dice coefficient metrics to assess any changes in model accuracy and reliability.

The analysis in Table 5 revealed that while there is a slight decrease in performance metrics with increased deviations in brightness and contrast, the FPN_resnet34 model maintained a robust performance profile. Specifically, the model demonstrated a decrease of only 5% in the Dice coefficient at the extreme ends of the adjustments, confirming its capability to handle typical clinical variations effectively.

**Table 5:** Comparison of best performing model on different brightness and contrast.

Dataset	Precision	Recall	Accuracy	Dice coefficient
Normal dataset	0.574105	0.678503	0.924901	0.790132
Adjusted brightness	0.446907	0.790718	0.887756	0.74354
Adjusted contrast	0.439643	0.825564	0.871064	0.737718

This finding is crucial for clinical applications, as it assures that the FPN_resnet34 model can be reliably used in diverse cystoscopy setups, accommodating common variations in image quality without significant loss of accuracy. It supports the model’s suitability for integration into clinical workflows, where it can aid in the reliable detection and segmentation of TCC polyps, enhancing diagnostic processes even under less-than-ideal imaging conditions.

## 4 Discussion

Our investigation has spanned a range of deep learning architectures, each rigorously tested for the segmentation of transitional cell carcinoma (TCC) polyps in cystoscopy images. The Unetplusplus_vgg19, serving as our study’s benchmark, not only achieved a precision of 55.40% and recall of 79.97% but also demonstrated the principle of “Clinical Integration.” This principle is central to our research, underscoring the capability of these models to be deployed within existing medical workflows, complementing standard diagnostic procedures without necessitating systemic changes or additional training for medical staff.

Building on this foundation, models like Unet_vgg11 and PSPNet_vgg11 revealed their strengths in recall, highlighting their efficacy in polyp detection against the backdrop of variable image quality—a common challenge in clinical environments. The high recall rates, reaching up to 87.87% with PSPNet_vgg11, are indicative of the models’ potential utility in emergency or urgent care settings where the timely identification of malignancies can be crucial. However, the precision of these models points to the need for continued refinement to balance sensitivity with specificity.

Addressing the trade-offs between precision and recall, the MAnet_resnet34 and Linknet_inceptionv4 architectures have shown that deep learning can adapt to the nuanced demands of medical image analysis. FPN_resnet34, in particular, with the highest precision among the models evaluated, exemplifies the type of targeted performance that can significantly impact clinical decision-making, especially in scenarios where the accuracy of polyp identification is critical, and false positives can carry significant risks.

The ROC curves, with their illustrative AUC values, provide a visual testament to the classification prowess of these models. The performance on lower-quality images, a testament to their robustness, enhances the prospect of these models being clinically integrated into diverse healthcare settings, including those with resource constraints. It showcases the potential of AI to democratize access to advanced diagnostic tools, thus broadening the scope of AI-driven healthcare.

In summary, our deep learning models have demonstrated that they can substantially improve the segmentation of TCC polyps, even in the face of challenging imaging conditions. Their clinical integration promises to streamline the diagnostic process, enabling more accurate and expedient patient care. This research contributes to the burgeoning evidence that supports the adoption of AI in medical imaging and underscores the significance of selecting the right deep learning architecture to meet the specific requirements of medical imaging tasks. With the models’ clinical integration, they are set to become invaluable assets in the ongoing battle against bladder cancer, enhancing both the efficacy and efficiency of patient care in the field of urology.

## 5 Conclusion

In this paper, we have delved into the potential of deep learning models to segment TCC polyps in cystoscopy images, a task both critical and challenging, particularly when images are of suboptimal quality. Our exploration into the capabilities of models like Unetplusplus_vgg19 and FPN_resnet34, trained on these low-quality images, has yielded results that are not only promising but also practically applicable in current medical procedures.

The models demonstrated varying degrees of precision and recall, with Unetplusplus_vgg19 establishing a solid baseline with 55.40% precision and 79.97% recall, and FPN_resnet34 showing a high precision of 57.41%. Despite the challenges posed by the image quality, these models have managed to perform with notable accuracy, as evidenced by their respective Dice coefficients.

Crucially, the application of these models does not necessitate any modifications to existing clinical processes. This underscores their practicality and potential for seamless integration into current diagnostic workflows, thereby minimizing disruption in clinical settings and facilitating a smoother transition towards AI-augmented medical practices.

Furthermore, the fact that these models have been trained on lower-quality images—a common occurrence in many clinical environments—emphasizes their robustness and adaptability. It also highlights a critical aspect of medical AI deployment: the ability of models to generalize and maintain performance despite less-than-ideal input conditions.

Looking ahead, the capacity for these models to be used in real-time clinical applications presents an exciting avenue for future research. The prospect of integrating these models into live diagnostic procedures could greatly assist medical professionals by providing real-time insights and augmenting their decision-making processes particularly in detecting suspicious lesions, and the more accurate visual perception of tumor grade.

In conclusion, our study not only showcases the significant strides made in applying deep learning to medical image analysis but also stresses the importance of developing AI tools that are compatible with the realities of clinical practice. As we advance, it remains imperative to ensure that these innovations align with the nuanced needs of healthcare providers, enabling them to deliver the highest standard of patient care. The potential of deep learning to revolutionize the field of healthcare is immense, and our research is a testament to its transformative impact, particularly in the early detection and treatment of bladder cancer.

## Data availability statement

The raw data supporting the conclusions of this article will be made available by the authors, without undue reservation.

## Ethics statement

The studies involving humans were approved by Research Ethics Committees of Tarbiat Modares University. The studies were conducted in accordance with the local legislation and institutional requirements. The anonymized codified images used in this study were acquired from the intraoperative image streaming. Written informed consent for the use of anonymized data was aquired from the participants or the participants’ legal guardians/next of kin in accordance with the national legislation and institutional requirements.

## Author contributions

M-RB: Writing – original draft, Writing – review & editing, Conceptualization, Data curation, Investigation, Methodology. MS: Supervision, Project administration, Writing – review & editing. PS: Supervision, Conceptualization, Validation, Writing – review & editing. FK: Data curation, Validation, Writing – review & editing.
